# Direct formation of peritectic phase but no primary phase appearance within Ni_83.25_Zr_16.75_ peritectic alloy during free fall

**DOI:** 10.1038/srep22641

**Published:** 2016-03-03

**Authors:** P. Lü, H. P. Wang

**Affiliations:** 1 MOE key Laboratory of Space Applied Physics and Chemistry, Department of Applied Physics, Northwestern Polytechnical University, Xi’an 710072, P. R. China

## Abstract

Ni_83.25_Zr_16.75_ peritectic alloy was containerlessly solidified in a drop tube. When the droplet diameter exceeds a critical value (*D*_crit_), Ni_7_Zr_2_ phase primarily solidifies, followed by the peritectic reaction of Ni_7_Zr_2_ + L → Ni_5_Zr. Once the droplet diameter is smaller than the critical value (*D*_crit_), peritectic phase Ni_5_Zr directly solidifies from the undercooled melt by completely suppressing the nucleation and growth of Ni_7_Zr_2_ phase, which is ascribed to high undercooling and cooling rate. Additionally, peritectic phase Ni_5_Zr grows equiaxially in the sample solidified in a DSC at a cooling rate of 0.167 K/s.

Peritectic reaction, in which the primary phase reacts with a liquid phase at a triple junction on cooling to produce the peritectic phase, is observed in many binary alloys systems, such as Ti-Al, Fe-Co, Fe-Ni, etc^1^,^2^,^3^. Peritectic reaction terminates once the primary phase is enwrapped by the peritectic phase. Then, the primary phase transforms to the peritectic phase by peritectic transformation. Due to the sluggishness of long-range solid-state diffusion, peritectic transformation could not proceed completely, leading to the existence of primary phase in the final microstructure after peritectic solidification. Recently, microstructures containing only peritectic phase with no primary phase were reported during the solidification of highly undercooled peritectic alloys, which has aroused great interest^4^,^5^,^6^,^7^,^8^,^9^. Wei *et al*.^6^ reported that once the undercooling exceeds a critical value of about 220 K, peritectic phase forms directly from the metastable liquid phase by suppressing the nucleation of primary phase for Cu-70%Sn alloy, which is hyperperitectic composition. Phanikumar *et al*.^8^ found that the microstructure is nearly phase-pure peritectic phase when Fe-25%Ge peritectic alloy is undercooled up to 110 K using electromagnetic levitation technique, in which the primary phase is a solid solute phase of α-Fe. Löser *et al*.^9^ reported that beyond a critical undercooling, the equilibrium solidification can be replaced by the direct growth of peritectic phase for Co_75_Si_25_ peritectic alloy. Although some study results have been reported, fundamental and deep understanding of different aspects of peritectic solidification is still poor. These investigations mainly focus on the effect of undercooling on phase selection and microstructure evolution. It is not clear how the peritectic phase could directly form from liquid alloys, especially for some complicated peritectic alloys, for example, both the primary phase and the peritectic phase are intermetallic compounds.

The Ni-Zr binary alloy system contains abundant metallic compounds and amorphous alloys, which have been studied extensively^10^,^11^,^12^,^13^,^14^,^15^,^16^. However, most works concentrate on the atomic structure and glass forming ability. Ni_83.25_Zr_16.75_ alloy is a typical peritectic composition in Ni-Zr binary alloy system. The primary phase Ni_7_Zr_2_ and peritectic phase Ni_5_Zr are both intermetallic compounds. Phase selections between primary phase and peritectic phase have great influence on the final solidified microstructures, which directly relate to the materials characteristics. Therefore, the objective of this work is to investigate effects of undercooling and cooling rate on phase selection and microstructure evolution of Ni_83.25_Zr_16.75_ peritectic alloy by a 3 m drop tube. Meanwhile, the effect of cooling rate on peritectic growth is also studied.

## Results and Discussion

**Figure 1:**
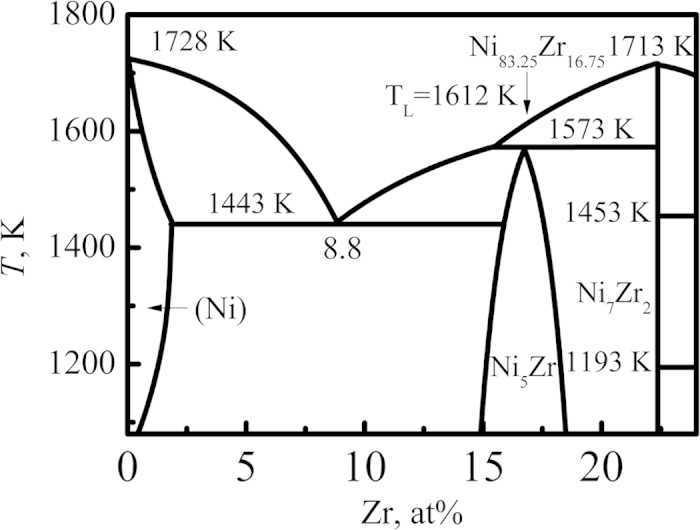
The left part of Ni-Zr binary phase diagram^17^.

Figure 1 shows the left part of Ni-Zr binary phase diagram^17^, in which the studied Ni_83.25_Zr_16.75_ peritectic alloy is marked with an arrow. As can been seen, the solidification of Ni_83.25_Zr_16.75_ peritectic alloy starts with the formation of primary Ni_7_Zr_2_ phase and a peritectic reaction of Ni_7_Zr_2_ + *L* → Ni_5_Zr occurs at 1573 K under the equilibrium condition. 100% peritectic phase is obtained when peritectic solidification is accomplished.

**Figure 2:**
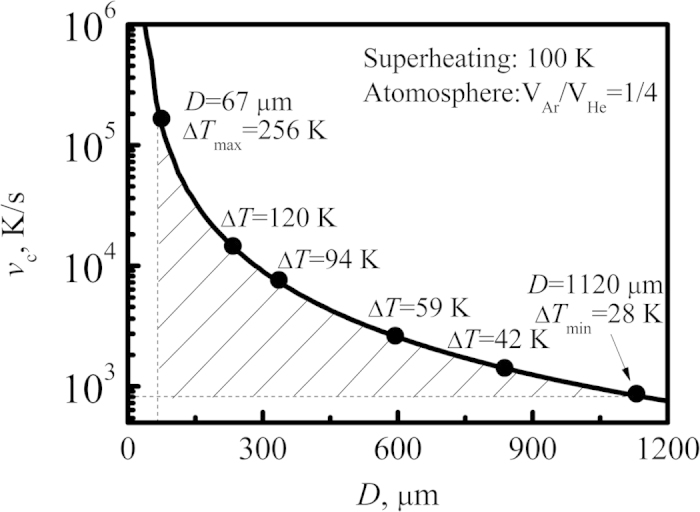
Calculated average cooling rate and undercooling versus droplet diameter.

Drop tube processing has the advantage to achieve high cooling rate and undercooling, which is quite suitable to investigate the phase selection. However, it is hard to measure and record the temperature experimentally due to the short time of free fall. Under this condition, heat transfer theory^18^,^19^ is applied to calculate the cooling rate and undercooling of droplets. The calculated results are shown in Fig. 2, and Table 1 lists the physical parameters used in the calculation^20^. It can be seen that the cooling rate and undercooling strongly depend on droplet diameter.

**Figure 3:**
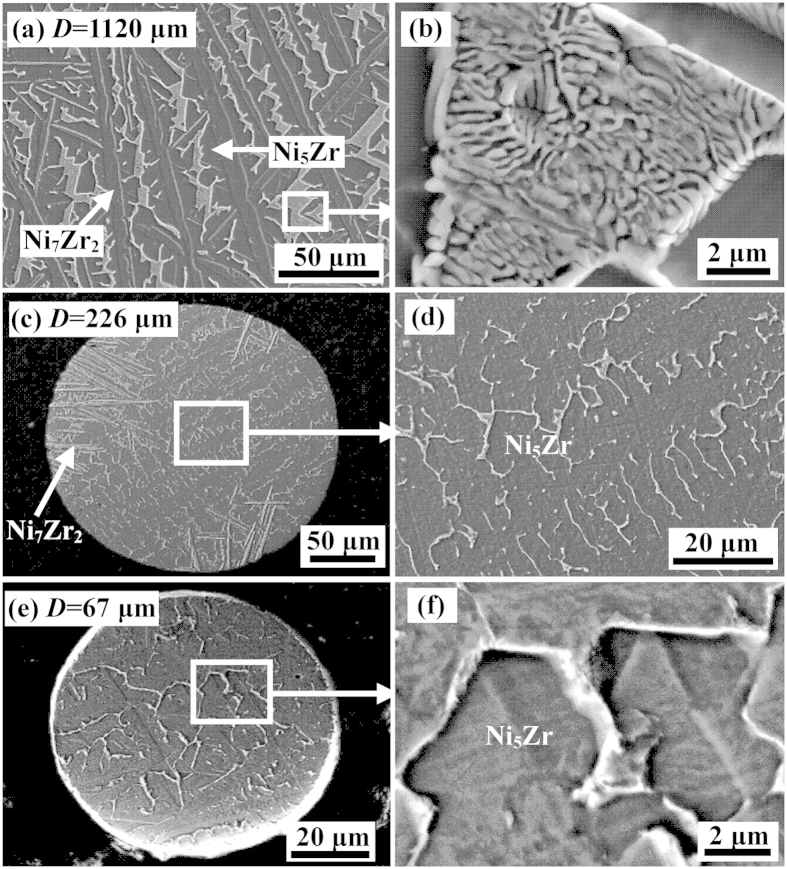
Solidified microstuctures of Ni_83.25_Zr_16.75_ peritectic droplets with different diameters. (**a**,**b**) *D* = 1120 μm; (**c, d**) *D* = 226 μm; (**e**,**f**) *D* = 67 μm.

Figure 3 illustrates the solidified microstructures of Ni_83.25_Zr_16.75_ peritectic droplets with different diameters, in which the primary phase Ni_7_Zr_2_ and the peritectic product Ni_5_Zr have been marked. In the droplet of 1120 μm, the solidified microstructures are composed of primary Ni_7_Zr_2_ dendrites, peritectic phase Ni_5_Zr and inter-dendritic eutectic, as show in Fig. 3(a). Evidently, the primary phase Ni_7_Zr_2_ is characterized by coarse, developed dendrites and surrounded by peritectic phase Ni_5_Zr. Figure 3(b) is the enlargement of inter-dendritic eutectic microstructure in Fig. 3(a). It must be noticed that there is no any prediction of eutectic transformation for this composition according to the equilibrium phase diagram. This will be discussed in the following parts. With the decrease of droplet diameter, the refinement and fragment of primary Ni_7_Zr_2_ dendrites occur. When the droplet diameters decrease to 226 μm, the microstructure consists of two regions, as presented in Fig. 3(c). Clearly, one is primary Ni_7_Zr_2_ dendrites surrounded by peritectic phase Ni_5_Zr, which locate at the rim of the droplet. The other is Ni_5_Zr phase with no primary phase Ni_7_Zr_2_ locating at the center of the droplet, as illustrated in Fig. 3(d). The peritectic phase Ni_5_Zr is the predominant phase. When the droplet diameter is very small like 67 μm, only peritectic phase Ni_5_Zr grows within the whole droplet and no Ni_7_Zr_2_ dendrites can be observed, as presented in Fig. 3(e,f).

According to Kerr and Kurz’s description^21^, peritectic solidification consists of two processes, one is the solidification of primary phase and the other is the growth of peritectic phase. Meanwhile, three stages have been identified during a peritectic growth process, namely, peritectic reaction, peritectic transformation, and direct solidification of the peritectic phase. Different solidification pathway of Ni_83.25_Zr_16.75_ peritectic droplets can be concluded based on the solidified microstructure presented in Fig. 3. In the droplets whose diameters are large, the primary phase Ni_7_Zr_2_ is expected to nucleate at temperature below the liquidus temperature and grows into the manner of dendrites. With the decrease of droplets temperature, the peritectic phase Ni_5_Zr starts to nucleate at the surface of primary phase Ni_7_Zr_2_ when the temperature below the peritectic temperature. The requirement of peritectic reaction, which is primary phase, peritectic phase and liquid must be in contact with each other at a triple junction, is satisfied after the nucleation of peritectic phase Ni_5_Zr. Then, peritectic reaction starts and peritectic phase Ni_5_Zr grows along the surface of primary Ni_7_Zr_2_ dendrites to form a thin layer. Since peritectic reaction is controlled by short-range atomic diffusion in the liquid ahead of primary Ni_7_Zr_2_ dendrites and peritectic phase Ni_5_Zr, it is able to accomplish rapidly at the initial stage of the peritectic growth process^21^. Once the primary Ni_7_Zr_2_ dendrites are enveloped by the peritectic phase, the primary phase and liquid are separated by the peritectic phase, which results in the disappearance of the triple junction and the termination of peritectic reaction. After peritectic reaction, the peritectic phase Ni_5_Zr grows into the primary Ni_7_Zr_2_ dendrites by peritectic transformation and into the liquid by direct solidification. Peritectic transformation is controlled by long-range solid-state diffusion and the cooling rate in drop tube processing is always high, leading that peritectic transformation could hardly take place. Therefore, the primary Ni_7_Zr_2_ dendrites are retained in the microstructure, as illustrated in Fig. 3(a). Simultaneously, peritectic phase Ni_5_Zr grows into the liquid by direct solidification, which results in the deviation of residual liquid composition. As a consequence, the residual liquid solidifies as eutectic when the temperature decreases below the eutectic temperature, as illustrated in Fig. 3(b). The morphology in Fig. 3(b) is characterized by lamellar eutectic structure, where the bright one is (Ni) phase and the other is Ni_5_Zr phase.

**Figure 4:**
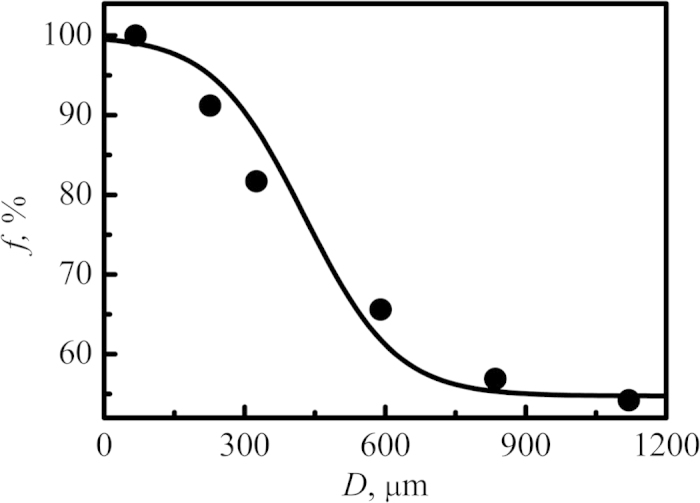
Average volume fractions of peritectic phase Ni_5_Zr versus droplet diameter.

With the decrease of droplet diameters, the possibility for a droplet to contain heterogeneous nucleation site is reduced. Therefore, droplets with small diameters can obtain high undercoolings. The enhancement of undercooling greatly promotes the nucleate rate of primary phase Ni_7_Zr_2_, which results in the refinement of primary Ni_7_Zr_2_ dendrites. Meanwhile, the release of latent heat remelts the primary phase leading to the fragment of Ni_7_Zr_2_ dendrites. As a result, the contact area between primary Ni_7_Zr_2_ dendrites and liquid increases. This apparently promotes the peritectic reaction and thus more primary Ni_7_Zr_2_ dendrites are decomposed. Similarly, residual liquid directly solidifies as Ni_5_Zr phase and a small amount of eutectic successively. According to the above analysis, it can be speculated that the volume fractions of peritectic phase increase with the decrease of droplet diameters. In order to validate the conclusion, the volume fractions of peritectic phase have been measured, which is illustrated in Fig. 4. Apparently, with the decrease of droplet diameters, the volume fraction of peritectic phase Ni_5_Zr increases, which is consistent with the speculation.

When the droplet diameter is 226 μm, the microstructure consists of only a small amount of primary Ni_7_Zr_2_ dendrites near the rim of droplet and predominant Ni_5_Zr phase without any Ni_7_Zr_2_ phase in the center, as presented in Fig. 3(c,d). The possibility for the formation of such a microstructure is that the peritectic phase Ni_5_Zr homogeneously nucleates at the center of the undercooled droplet and then starts to grow. Unfortunately, the peritectic phase Ni_5_Zr grows into a faceted way, the growth velocity of which is sluggish. The release of crystallization heat leads to a rise of temperature and a decrease of interface undercooling of Ni_5_Zr phase. Therefore, the growth of peritectic phase Ni_5_Zr will be terminated. At this small undercooling, the primary phase Ni_7_Zr_2_ is preferred to nucleate and grow. Following that, peritectic reaction takes place when the temperature drops below the peritectic temperature *T*_P_ . As for Ni_83.25_Zr_16.75_ peritectic alloy, the liquidus temperature of Ni_7_Zr_2_ phase is larger than that of Ni_5_Zr phase about 39 K, which leads to a high driving force for the nucleation of Ni_7_Zr_2_ phase. Therefore, Ni_7_Zr_2_ phase is preferred to nucleate. However, this is not the case under high undercooling condition. From a thermodynamic point of view, the change in Gibbs free energy, *dG*, determines which phase is thermodynamically stable at given temperature and pressure, expressed as


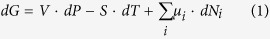


Equation (1) relates the change in Gibbs free energy to the sum of products of volume *V*, entropy *S*, chemical potential μ_*i*_ and the changes of pressure *dP*, temperature *dT* and number of particles *dN*_*i*_^22^. Chemical potential is the driving force of atom diffusion in a concentration field.

If the droplet is undercooled below the peritectic temperature *T*_P_ , the formation of a new phase is determined by the competitive nucleation of Ni_7_Zr_2_ phase and Ni_5_Zr phase. Clusters are the initial stage of nucleation. The formation of clusters needs atoms diffuse and attach together. When the attachment rate of atoms to the cluster is larger than the detachment rate of atoms to the same cluster, this cluster can form stable within the undercooled droplet. The concentration of droplet is the same as that of peritectic phase Ni_5_Zr. As a consequence, large chemical potential is required to form the clusters of Ni_7_Zr_2_ phase compared with the formation of Ni_5_Zr clusters, resulting in large Gibbs free energy barrier to nucleation of Ni_7_Zr_2_ phase. Thus, compared with the nucleation of Ni_5_Zr phase, high undercooling is required to nucleate the Ni_7_Zr_2_ phase. Fortunately, the undercooling of Ni_7_Zr_2_ phase Δ*T* is always larger than the undercooling of Ni_5_Zr phase Δ*T*_p_ about 39 K, which is beneficial to the nucleation of Ni_7_Zr_2_ phase. Therefore, Ni_7_Zr_2_ phase is still preferred to nucleate when the droplet is undercooled just below the peritectic temperature *T*_p_ . However, the Δ*T*/Δ*T*_p_ ratio is decreased with the further increase of undercooling Δ*T*, leading to the disappearance of the advantage of the nucleation of Ni_7_Zr_2_ phase. The undercooling is up to 120 K for the droplet whose diameter is 226 μm. Hence, peritectic phase Ni_5_Zr may primarily nucleates from the undercooled droplet when the droplet diameter is 226 μm.

With the further decease of droplet diameter, the peritectic phase Ni_5_Zr is more preferred to nucleate. A heat flux from the melt to the surroundings is required during the solidification process, which changes the free energies and therefore the relative thermodynamic stability, of the phases present^23^. Actually, the heat flux from the melt to the surrounding is absolutely dominated by the cooling rate. In the droplets whose diameter is 67 μm, the cooling rate is up to 1.8 × 10^5^ K/s, which leads that the crystallization heat is rapidly transformed to the surroundings during the peritectic growth. Therefore, the peritectic Ni_5_Zr phase can continually grow, resulting in the formation of phase-pure Ni_5_Zr microstructure, as shown in Fig. 3(e–f). The EDS analysis demonstrates that it is peritectic phase Ni_5_Zr which contains about 81 at% Ni and 19 at% Zr. This indicates that high undercooling and cooling rate may totally suppress the formation of Ni_7_Zr_2_ phase and results in the directly nucleation and growth of peritectic phase Ni_5_Zr from the undercooled melt. Besides, the critical diameter for obtaining phase-pure Ni_5_Zr microstructure is in the range of 226 to 67 μm.

**Figure 5:**
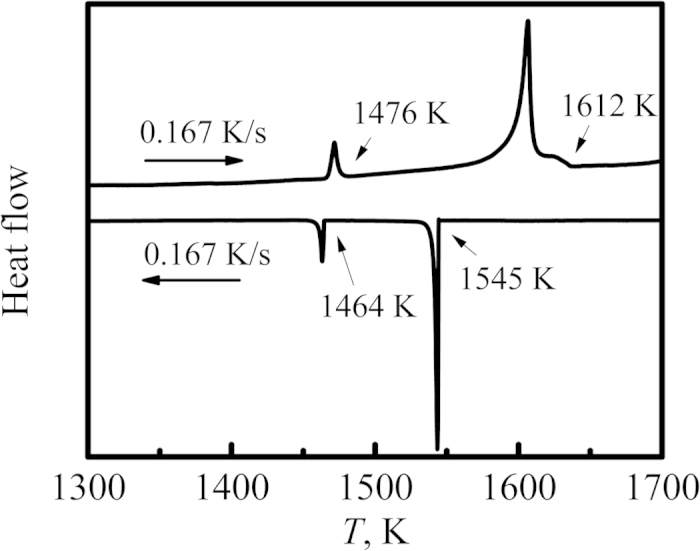
DSC curve of Ni_83.25_Zr_16.75_ peritectic alloy.

**Figure 6:**
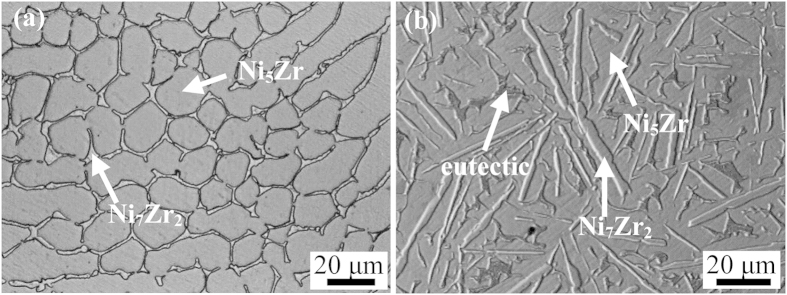
Microstructures of Ni_83.25_Zr_16.75_ sample solidified under different cooling rate. (**a**) cooling rate of 0.167 K/s in the DSC; (**b**) cooling rate of 2.8 × 10^3^ K/s in the drop tube.

In order to further investigate the effects of cooling rates on peritectic growth, DSC experiment was employed to perform a comparative analysis. 20 mg of Ni_83.25_Zr_16.75_ samples was completely melted by heating to 1723 K and then solidified at a cooling rate of 0.167 K/s. The DSC curves are shown in Fig. 5, in which the upper curve is the heating curve and the other is the cooling curve. It can be seen that there are two peaks during the solidification, which represent the solidification of primary phase Ni_7_Zr_2_ and the growth of peritectic phase Ni_5_Zr, respectively. The solidified sample has a undercooling of 67 K and the microstructure is illustrated in Fig. 6(a). Obviously, the microstructure consists of predominate peritectic phase Ni_5_Zr and a small amount of primary phase Ni_7_Zr_2_. Besides, a majority of peritectic phase Ni_5_Zr grows equiaxially. This indicates that peritectic transformation proceeds for a relatively long time duo to the low cooling rate in the DSC. Figure 6(b) shows the microstructure solidified in the drop tube at the similar undercooling level but with a high cooling rate of about 2.8 × 10^3^ K/s. The microstructure are composed of primary phase Ni_7_Zr_2_, peritectic phase Ni_5_Zr and eutectic, which shows completely different morphology compared with that in Fig. 6(a). The Ni_5_Zr phase exhibits faceted morphology, indicating that peritectic phase grows mainly by peritectic reaction and direct solidification under high cooling rate condition. This reveals a different peritectic growth mechanism under different cooling rate condition.

## Conclusion

In summary, Ni_83.25_Zr_16.75_ peritectic alloy was rapidly solidified in a 3 m drop tube. The microstructure evolution with the decrease of droplet diameter is investigated. For large droplets, solidified microstructures are composed of primary Ni_7_Zr_2_ dendrites, peritectic phase Ni_5_Zr and eutectic. The competitive nucleation and growth between Ni_7_Zr_2_ phase and Ni_5_Zr phase become intensive as droplet diameter decreases. The solidified microstructure consists of only peritectic phase Ni_5_Zr once the droplet diameter less than a critical value, *D*_crit_, which is determined in the range of 226 to 67 μm. For *D* > *D*_crit_, Ni_7_Zr_2_ phase primarily solidified, followed by the peritectic reaction of Ni_7_Zr_2_ + L → Ni_5_Zr. For *D* < *D*_crit,_ peritectic Ni_5_Zr phase directly solidifies from the undercooled melt by completely suppressing the nucleation and growth of Ni_7_Zr_2_ phase, which is ascribed to high undercooling and cooling rate. Ni_83.25_Zr_16.75_ sample solidified in the DSC contains no eutectic and the morphologies of peritectic phase Ni_5_Zr is equiaxial, indicating that peritectic phase grows mainly by peritectic reaction and peritectic transformation under low cooling rate condition.

## Experimental Details

Containerless rapid solidification of Ni_83.25_Zr_16.75_ peritectic alloy was performed in a 3 m drop tube. The master alloy samples were prepared by 99.99% pure Ni and 99.9% pure Zr mixtures in an argon atmosphere, and each of samples has a mass of 1.0 g. When the experiment began, the sample was placed in a silica tube, which has a *Φ*0.2 mm orifice at bottom and was installed at the top of 3 m drop tube. The drop tube was evacuated to 10^−5^ Pa and backfilled with 20% Ar and 80% He gas mixture to 1 atm. After melted by induction heating, the sample was ejected out from the orifice and dispersed into many fine droplets by exerting argon gas flow. The droplets were solidified rapidly in a containerless state during free fall. After that, the droplets were collected and sieved into several groups according to their sizes. The solidified samples were analyzed by FEI Sirion SEM and Oxford INCA 300 EDS.

## Additional Information

**How to cite this article**: Lü, P. and Wang, H. P. Direct formation of peritectic phase but no primary phase appearance within Ni_83.25_Zr_16.75_ peritectic alloy during free fall. *Sci. Rep.* **6**, 22641; doi: 10.1038/srep22641 (2016).

## Figures and Tables

**Table 1:** Physical parameters of Ni_83.25_Zr_16.75_ peritectic alloy^20^.

**Parameter**	**Value**
Peritectic composition, *C*_0_ (at.%)	16.75
Peritectic temperature, *T*_p_ (K)	1612
Heat of fusion, Δ*H* (J mol^−1^)	9176
Heat capacity, *C*_p_(J/mol/K)	36.84
Mass density, *ρ*(Kg m^−3^)	8092
Stefan-Boltzmann constant, *σ*_SB_(W cm^−2^ K^−4^)	5.67 × 10^−12^
Surface emissivity, *ε*_h_	0.45
